# Medical Society of the State of New York

**Published:** 1891-02

**Authors:** 


### Society Meetings.

### MEDICAL SOCIETY OF THE STATE OF NEW YORK.

#### OFFICERS AND COMMITTEES OF THE SOCIETY.

*President* – William Warren Potter, Buffalo. *Vice-President* — Lewis S. Pilcher, Brooklyn. *Secretary* — Frederic C. Curtis, Albany. *Treasurer* – Charles H. Porter, Albany.

*Committee of Arrangements* — Samuel B. Ward, Albany; Edward L. Partridge, New York; Cyrus S. Merrill, Albany. *Committee on By-Laws* — Laurence Johnson, New York; A. R. Simmons, Utica; F. C. Curtis, Albany. *Committee on Hygiene* — E. V. Stoddard, Rochester; A. N. Bell, Brooklyn; Chas. W. Hamlin, Middleville; J. P. Creveling, Auburn; Wm. H. Bailey, Albany; Wm. C. Bailey, Albion; E. F. Brush, Mt. Vernon. *Committee on Legislation* — D. B. St. John Roosa, New York; Daniel Lewis, New York; Maurice J. Lewi, Albany. *Committee*

*on Medical Ethics* – A. Jacobi, New York; Arthur Mathewson, Brooklyn; H. R. Hopkins, Buffalo. *Committee on Prize Essays*—George F. Shrady, New York; Daniel Lewis, New York; Eugene Beach, Gloversville. *Committee on Publication* – F. C. Curtis, Albany; Chas. H. Porter, Albany; Charles Cary, Buffalo; O. B. Douglas, New York.

Provisional programme of papers for the eighty-fifth annual meeting in the City Hall, at Albany, on Tuesday, Wednesday, and Thursday, February 3d, 4th, and 5th, 1891, commencing at 9.15 A. M. Tuesday, and ending at 5 P. M. Thursday.

*Business Committee*,

HERMAN BENDELL, 178 State St., Albany.

SENECA D. POWELL, 12 West 40th St., New York.

JAMES D. SPENCER, Watertown.

FIRST DAY—TUESDAY. MORNING SESSION – 9:15.

President's inaugural address, appointment of committees, executive business.

PAPERS.—Railroad Surgery, Clinton B. Herrick, Troy. A Report of Cases of Injury to the Knee-Joint, with Remarks, Henry Flood, Elmira. The Management of Sinuses in Chronic Bone and Joint Diseases, V. P. Gibney, New York. Necrosis of the Ribs, Complicating Potts' Disease, Louis A. Weigel, Rochester. Brief Notes on Gastrotomy, with Report of a Successful Case, Charles A. Powers, New York. The Action of Trypsin, Pancreatin, and Pepsin, upon Sloughs, Coagula and Mucopus, Robert T. Morris, New York. Operative Procedures in Acute General Suppurative Peritonitis, W. E. B. Davis, Birmingham, Ala. The progress of Cystoscopy in the last three years, Willy Meyer, New York. A Plea for Rapid Dilatation, Holt's Operation, in the Treatment of Urethal Stricture, F. R. Sturgis, New York. A Case of Imperforate Anus of Eight Weeks' Standing, Operation and Recovery, Wm. Hailes, Jr., Albany. A Contribution to the Surgical Treatment of Jacksonian Epilepsy—Excision of the Arm Center, Edward B. Angell, Rochester.

AFTERNOON SESSION – 2.15.

Diagnostic Significance of the State of the Pupils, E. C. Spitzka, New York. Three Diagnostic Symptoms of Melancholia—Second Communication, Landon Carter Gray, New York. Hysterical Manifestations Due to Alcoholism, H. C. Coe, New York. Cases of Traumatic Hysteria, Henry Hun, Albany. Insomnia and its Treatment, E. N. Brush, Philadelphia. The Causes of Asthenopia, D. B. St. John Roosa, New York. The Treatment of Detachment of the Retina, David Webster, New York. On the Use of Platinum Instruments in the Extraction of Cataract and in Other Operations upon the Eye, E. Gruening, New York.

Migraine and Headache from Eye Strain, Peter A. Callan, New York. One Thousand Cases of Ocular Headache and the Different States of Refraction Connected Therewith, W. F. Mittendorf, New York. Double Optic Neuritis, Frank W. Ring. New York. Some Points in the Pathogenesis of Aural Vertigo, Oren D. Pomeroy, New York. Catarrh and its Cure, O. B. Douglas, New York. The Correction of Angular Deformities of the Nose by a Subcutaneous Operation, John O. Roe, Rochester. Certain Hygienic Measures in the Treatment of Catarrhal Affections of the Upper Air Passages, F. H. Bosworth, New York.

### EVENING SESSION—7.15.

Discussion on Appendicitis.

The Pathology of Appendicitis, Herman Mynter, Buffalo; Arpad G. Gerster, New York. The Indications for Early Laparatomy in Appendicitis, Charles McBurney, New York; William W. Keen, Philadelphia. The Technique of Operative Interference in Appendicitis, Lewis A. Stimson, New York; George Ryerson Fowler, Brooklyn. The Propriety of and the Indications for the Resection of the Appendix Vermiformis During Quiescent Stage of Chronic Relapsing Appendicitis, Joseph Price, Philadelphia; Robert F. Weir, New York. The Relation of the Physician and the Surgeon in the Care of Cases of Appendicitis, Albert Vander Veer, Albany.

### SECOND DAY—WEDNESDAY. MORNING SESSION, 9.15.

Discussion on Pelvic Inflammation in Women: Its Pathology and its Palliative, Conservative and Radical Treatment.

Introduction, Andrew F. Currier, New York. Pathology, A. J. C. Skene, Brooklyn; W. Gill Wylie, New York. Palliative Treatment C. C. Lee, New York. Conservative Treatment, W. M. Polk, New York. Radical Treatment, Joseph Price, Philadelphia; L. S. McMurtry, Louisville, Ky.

These papers will be discussed by Dr. Thomas Addis Emmet (probably), of New York, Dr. Franklin Townsend, of Albany, and others.

PAPERS.—The Treatment of Posterior Displacements by the Utero-Vaginal Ligature, H. J. Boldt, New York. Minor Gynecological Surgery, Maurice J. Lewi, Albany. The Treatment of Injuries to the Floor of the Vagina, Horace Tracy Hanks, New York. An Inquiry into Our Present Knowledge of the Progress of Myomatous Tumors after (*a*) The Use of the Electric Current; (*b*) Removal of Ovaries and Tubes; (*c*) The Old Method of Treatment by Rest, Intrauterine Applications and Ergot, James F. W. Ross, Toronto. My Experience with the Surgical Treatment of Retroflexion, and Prolapsus Uteri, Paul F. Mundé, New York. Surgical Treatment of Ectopic Gestation, Charles A. L. Reed, Cincinnati. O. Some of the Results of Defective Sanitary Arrangements in the Puerperal State, James P. Boyd, Albany. The Management of Tedious Labor, Egbert H. Grandin, New York. A Report of Four Cases of Cancer of the Clitoris where Clitoridectomy was Performed, Franklin Townsend, Jr., Albany, The Emotional Element : the Puerperal Period, Adam H. Wright, Toronto.

AFTERNOON SESSION—2.15.

Discussion on Pulmonary Tuberculosis.

History, H. R. Hopkins, Buffalo. Etiology and Pathology, Heneage Gibbes, Ann Harbor, Mich. Diagnosis and Prognosis, Alfred L. Loomis, New York. Manifestation in Upper Air Tract and Special Treatment thereof, John O. Roe, Rochester. Treatment, including Prophylaxis, as Related to Climate, Samuel B. Ward, Albany. Treatment as Related to Therapeutics, E. L. Shurley, Detroit.

PAPERS.—Koch's Lymph, and Tuberculosis, A. Jacobi, New York. Experiences at the Mt. Sinai Hospital and Polyclinic Hospital with Koch's Lymph, Henry N. Heineman, New York. Therapeutical Notes on Acute Respiratory Diseases, A. Jacobi, New York. Lobar Pneumonia with the Production of Connective Tissue in the Air Spaces, Francis Delafield, New York. The Treatment of Gall Stones, William Wotkyns Seymour, Troy. Treatment of Itching, Edward B. Bronson, New York. On the Causes of Eczema, L. Duncan Bulkley, New York. A Case of Pemphigus, H. R. Hopkins, Buffalo, The Treatment of Pigmentary and Vascular Naevi, George Henry Fox, New York.

EVENING SESSION.

Anniversary Address by the President, eight o'clock, Assembly Chamber. Annual dinner of the Society, 9.30 o'clock, Delavan House.

THIRD DAY—THURSDAY. MORNING SESSION, 9.15.

Executive Business.

PAPERS.—How a Mammary Amputation for Cancer Should be Conducted, Robert F. Weir, New York. Electro-Cautery in Surgery, with Special Reference to its Use in the Nose, Mouth and Throat, D. H. Goodwillie, New York. Etiology and Development of Malignant Neoplasms, A. B. Macallum, Toronto. Intestinal Obstruction ; Report of Cases, J. H. Glass, Utica. The Unrestricted Evils of Prostitution, Andrew F. Currier, New York. Croupous Rhinitis, Frank H. Potter, Buffalo. Vesicular Eruption in Scarlatina, F. C. Curtis, Albany.

AFTERNOON SESSION—2.15.

The Relation of Physicians to Boards of Health, Lewis Balch, Albany. The Necessity of an Amendment in the Laws Governing Medical Evidence in Malpractice Suits, R. J. Wilding, Malone. The Present Status of the Proposed Law to Regulate the Practice of Embalming Human Dead Bodies, A. Walter Suiter, Herkimer. Subject to be announced, William S. Ely, Rochester. Subject to be announced, Clarence C. Rice, New York. Subject to be announced, Lucien Howe, Buffalo.

